# Use of carbon fiber-reinforced PEEK cages in spinal oncology patients: An institutional experience with emphasis on surgical, complication and imaging characteristics

**DOI:** 10.1007/s00701-025-06739-6

**Published:** 2025-12-20

**Authors:** Stefanos Voglis, Lina-Elisabeth Qasem, Leonhard Mann, Fatma Kilinc, Daniel Jussen, Matthias Setzer, Fee Keil, Vincent Prinz, Marcus Czabanka

**Affiliations:** 1 Department of Neurosurgery, University Hospital Frankfurt, Frankfurt, Germany; 2 Department of Neurosurgery, University Hospital Zurich, Zurich, Switzerland; 3 Institute of Neuroradiology, University Hospital Frankfurt, Frankfurt, Germany

**Keywords:** CFRP, Vertebral body cage, PEEK, Spine, Oncology

## Abstract

**Purpose:**

Radiolucent carbon fiber-reinforced PEEK (CFRP) pedicle screws have improved imaging and radiation planning in spine oncology. However, these benefits are often limited by titanium cages used in combination. This study reports our initial experience using novel CFRP cages in oncologic spine surgeries, comparing their surgical feasibility, complications, and imaging performance to titanium cages.

**Methods:**

We retrospectively analyzed 13 patients who received CFRP cages between July 2021 and May 2023. Clinical, surgical, follow-up, and imaging data were evaluated. A matched cohort with titanium cages was used to assess postoperative MRI visibility of anatomical landmarks, rated by independent reviewers.

**Results:**

Thirteen CFRP cages were implanted (mean age 61 ± 11 years; 54% female). Indications included spinal metastases (54%) and primary bone tumors. Most procedures involved the thoracic spine (62%), followed by lumbar (23%) and cervical (15%). Median instrumentation spanned 4 segments; 62% had two-staged surgeries. Three complications occurred: 1 cage dislocation (requiring revision), 1 wound infection, and 1 post-incisional hernia. Median radiologic follow-up was 74 days, with no further dislocations. Postoperative MRI showed significantly better visibility of key spinal landmarks with CFRP cages versus titanium (*p* < .05, Wilcoxon signed-rank test).

**Conclusion:**

CFRP cages are a viable alternative to titanium in oncologic spine surgery, offering comparable usability and improved postoperative imaging. These advantages may support better radiotherapy planning and complication detection, warranting further investigation.

**Supplementary Information:**

The online version contains supplementary material available at 10.1007/s00701-025-06739-6.

## Introduction

Carbon fiber-reinforced PEEK (CFRP) pedicle screws in oncologic spine surgery have proven to be a surgically feasible and safe alternative to titanium screws [7]. Such CFRP screws have increasingly being used in spinal oncology with the perceived advantages of postoperative imaging quality and therefore radiation planning, while maintaining the same results in terms of complications and surgical applicability [1, 3, 10, 11, 15].

Recent studies could show notable advantages in terms of their radiolucency and thus less interference with postoperative imaging modalities by reducing artifacts [3, 6] and thereby allowing a more precise postoperative monitoring and radiotherapy treatment planning [5, 7]. However, the clinical integration of titanium vertebral body cages with CFRP pedicle screws has often diminished the imaging advantages of these radiolucent screws.

In this regard, recent single-center [1, 13] and multi-center [2] studies which included among other CFRP implants newly available vertebral body cages, have shown that the use of these CFRP cages is not only surgically feasible, but also associated with a complication rate comparable to that of titanium cages in oncologic spine surgery. However, studies focusing on CFRP cages remain scarce. Additionally, the interpretability of postoperative MRI scans in patients with CFRP cages has not been evaluated in comparison to conventional titanium cage implants.

In this study, we present our initial experience using novel CFRP vertebral body cages in spinal oncology patients. We compare these cages to traditional titanium cages, focusing on key aspects such as surgical applicability, complication rates, and characteristics in postoperative imaging assessments.

## Methods

### Study cohort, data acquisition and ethical considerations

All patients who underwent implantation of a CFRP cage for oncological indications between August 2021 and May 2023 at the Department of Neurosurgery, University Hospital Frankfurt, Germany were included. For each patient, demographic, clinical and tumor characteristics as well as postoperative surgical complication, clinical and imaging follow-up outcomes were extracted from the electronic patient record according to local ethic board regulations.

### Scoring of postoperative MRI

To compare the interpretability of postoperative MRI in patients who underwent CFRP cage implantation, a matched cohort of patients who underwent titanium vertebral body implantation with similar demographic and surgical characteristics was assembled. MRI scoring was performed independently by experienced neuroradiologists (KF, ML) and neurosurgeons (QL, VS, PV) on sagittal and axial T2-weighted images. An arbitrary scoring system of 1–3 was used to evaluate the assessability (1: good, 2: limited, 3: no assessability) at different anatomical landmarks (anterior vertebral column, posterior vertebral column, neuroforamen, spinal canal, see Supplementary Fig. 1) at the level of the cage-implant. Kendall's coefficient of concordance W was used to assess interobserver agreement [8].

### Statistical analysis

All data processing and statistical analyses were performed using R (version 4.4.1) [12] and R Studio (version 2024.12.1, Posit Software) [14] and open-source libraries. Patient and surgical characteristics are presented as percentages (for categorical variables) and as median [IQR] or mean [SD] for continuous variables. Non-parametric tests were performed using either Fisher’s exact test (categorical variables) or Wilcoxon rank sum test (continuous variables), as indicated in the tables and figure legends. Missing values were considered as missing at random and therefore omitted from the analyses. P values < 0.05 were considered statistically significant. As the statistical analysis was exploratory, no correction for multiple testing was applied. Anonymized raw data and analysis scripts are available from the corresponding author upon request.

## Results

### Study cohort characteristics

A total of 13 CFRP cages were implanted in 13 patients between July 2021 and May 2023. Seven (54%) patients were female, and the mean age was 61 years [± 11 SD]. The majority of patients had vertebral metastases (*n* = 7, 54%), most commonly from breast cancer (*n* = 3), followed by primary bone tumors (most commonly plasma cell myeloma *n* = 3 and spinal hemangioma *n* = 2; see Table 1 for demographic and tumor characteristics).

**Table 1:** Patient and tumor characteristics

Characteristic	*N* = 13^*1*^
Sex	
Female	7 (54%)
Male	6 (46%)
Age	61 (11)
Tumor/Metastases	
Metastases	7 (54%)
Primary bone tumor	6 (46%)
Tumor entity	
Breast cancer	3 (23%)
Plasma cell myeloma	3 (23%)
Hemangioma	2 (15%)
Coloncarcinoma	1 (7.7%)
Melanoma	1 (7.7%)
Renal cell carcinoma	1 (7.7%)
Esophageal carcinoma	1 (7.7%)
Sarcomatoid tumor	1 (7.7%)

^*1*^ *n* (%), Mean (SD)

### Surgical characteristics

**Fig. 1:**
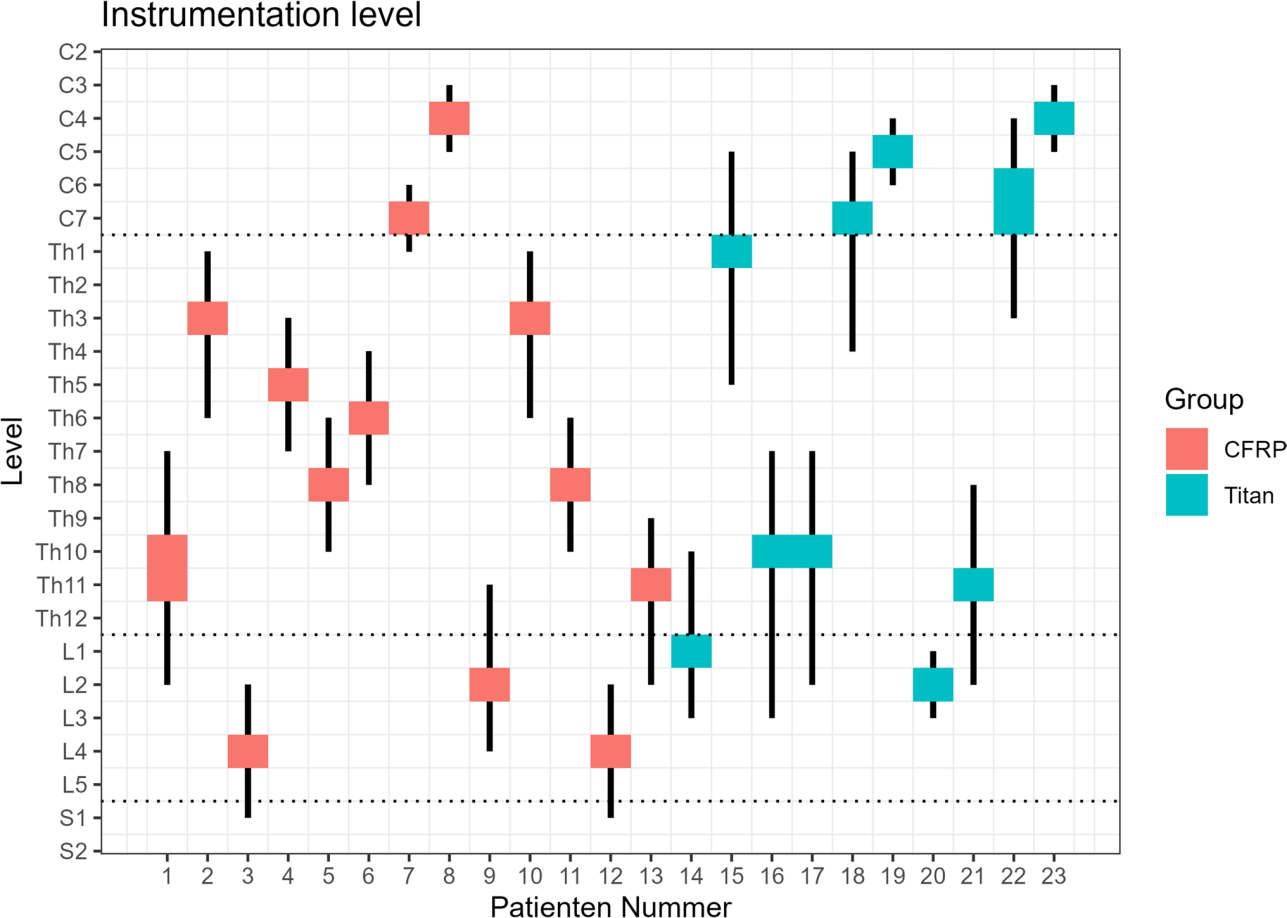
**Distribution instrumentation levels:** Level of instrumentation and vertebral body replacement for the respective patients. Vertical lines correspond to the extent of instrumentation, colored boxes to the vertebral body replacement. For example, patient no. 1 has had a CFRP cage implanted to bridge Th10 and 11

Vertebral body replacements with CFRP cages were most commonly performed in the thoracic spine with 62% of cases. This was followed by the lumbar spine with 23% of cases, the cervical spine with 15% of cases, and the comparison group with titanium cages with 40% of cases each in the thoracic and cervical spine and 20% in the lumbar spine (Table 2 and Fig. 1 for the spine distribution and the instrumented level for each case). The median length of the instrumentation was 4 segments for CFRP cages and 6 segments for titanium cages (Table 2, Fig. 1) and surgery was performed in a two-stage approach in most of the cases (62% for CFRP and 70% for titanium cages, Table 2).

**Table 2:** Surgical, complication and follow-up characteristics

Characteristic	Carbon *N* = 13^*1*^	Titan *N* = 10^*1*^	*p*-value^*2*^
Staged surgery			> 0.9
Two-stage	8 (62%)	7 (70%)	
Single-stage	5 (38%)	3 (30%)	
Number segments	4.00 (4.00, 5.00)	6.00 (2.00, 7.00)	0.2
Spine segment			0.5
Thoracic	8 (62%)	4 (40%)	
Cervical	2 (15%)	4 (40%)	
Lumbar	3 (23%)	2 (20%)	
Surgical complications			0.7
No	10 (77%)	6 (60%)	
Yes	3 (23%)	4 (40%)	
FU days	73.92 (133.75)	126.71 (119.34)	0.043
Unknown	0	3	

^*1*^ *n* (%), Median (Q1, Q3), Mean (SD)

^*2*^ Fisher’s exact test, Wilcoxon rank sum test

### Surgical complications and radiological follow-up

Surgical complications were observed in 3 patients with CFRP implants (vs. 4 in the titanium implant group, see Table 2). One case involved cage dislocation requiring revision surgery. Another patient developed a wound infection, and a third patient developed a post-incisional hernia. The mean radiologic follow-up was 74 days for CFRP implant patients with no additional cage dislocations (Table 2).

### Post-operative MRI assessability

**Fig. 2:**
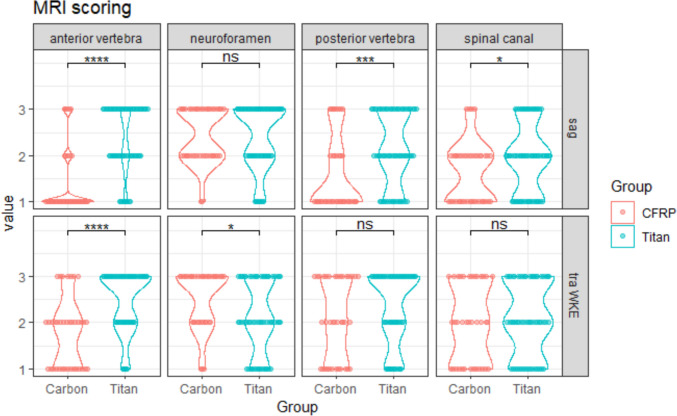
**MRI Scoring:** Subjective scoring of CFRP vs. titan cages vertebral body cages at different anatomical landmarks (column) in sagittal and transverse T2-weighted MRI sequences (row). 1 = can be assessed well 2 = limited 3 = cannot be assessed. Wilcoxon rank sum test

Postoperative MRI scoring at different anatomic landmarks was performed by independent neuroradiologists and neurosurgeons (Methods). To assess the interobserver agreement of the subjective scoring, Kendall's coefficient of concordance W was calculated and showed "substantial agreement" (0.6 ≤ w < 0.8) in all but two anatomic landmarks (anterior vertebral body on transverse sequences and neuroforamen on sagittal sequences with "moderate agreement", 0.4 ≤ w < 0.6) [8]. This scoring revealed significantly better visualization in the CFRP cage group compared to the titanium cage group (Fig. 2). Specifically, there was better visualization of the anterior/posterior vertebral body level and the spinal canal on sagittal T2 sequences, and the anterior vertebral body level and the neuroforamen level on axial T2 sequences (Fig. 2, Wilcoxon signed-rank test *p* < 0.05).

### Illustrative cases

#### Case 1 – cervical CFRP cage

**Fig. 3:**
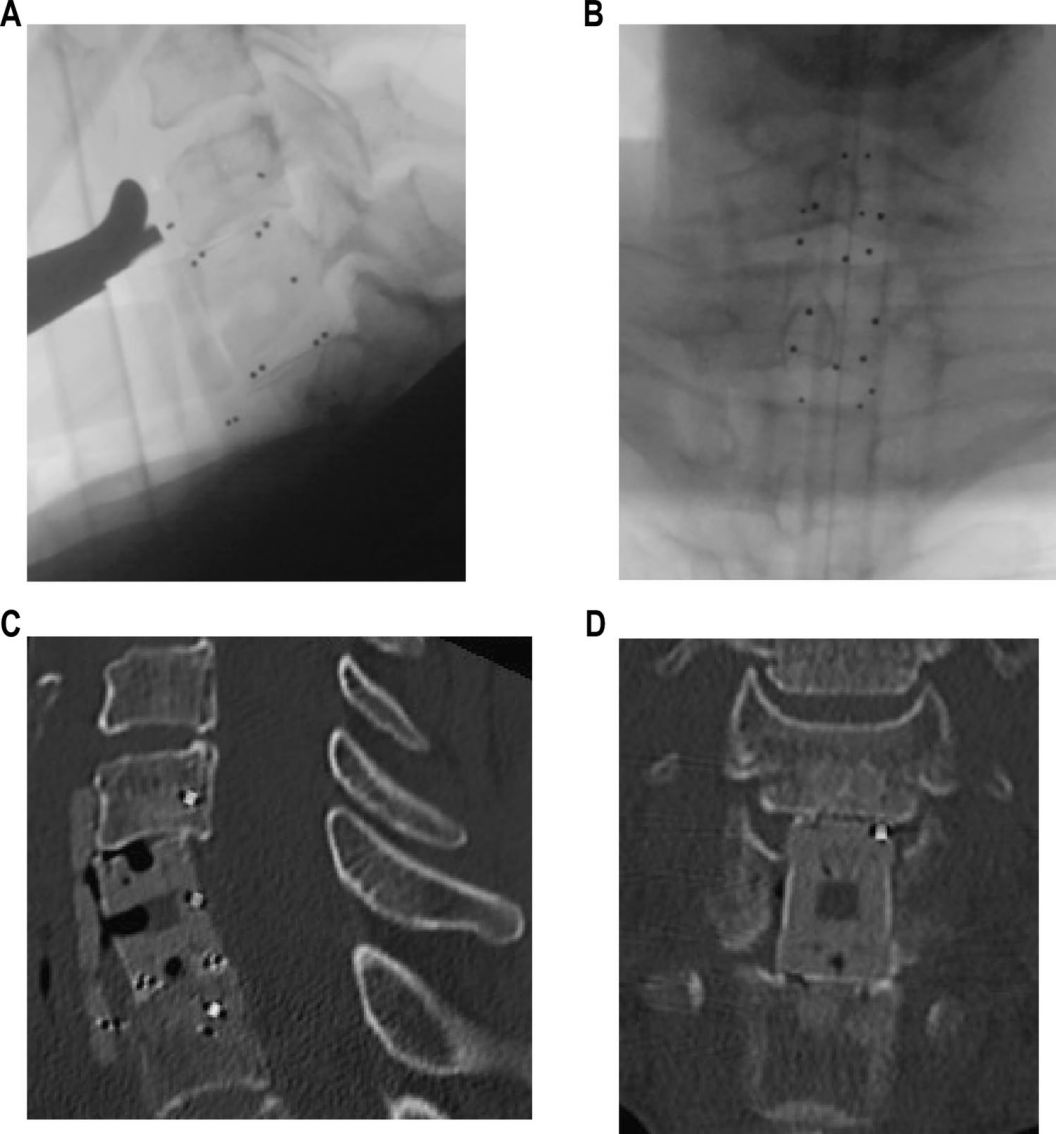
Case 1: Lateral (**A**) and a.p. (**B**) intraoperative X-ray controls after implantation of the CFRP cage. Postoperative CT controls in sagittal (**C**) and coronal view (**D**)

A 38-year-old female patient with spinal metastases from breast carcinoma, presenting with osteolytic destruction and a pathological fracture of C7, reported severe movement-dependent pain. Given the instability and pain-induced immobility, surgical intervention was performed. The patient underwent a single-stage ventral corpectomy with CFRP vertebral body replacement and ventral CFRP plating to restore spinal stability and to prevent spinal cord compression (intraoperative x-rays in Fig. 3A, B). A postoperative CT scan confirmed the correct positioning of the implants (Fig. 3C, D), and the patient recovered well from surgery. She subsequently received adjuvant radiochemotherapy as part of her oncological treatment plan.

#### Case 2 – cervical CFRP cage with postoperative MRI to rule out hemorrhage

**Fig. 4:**
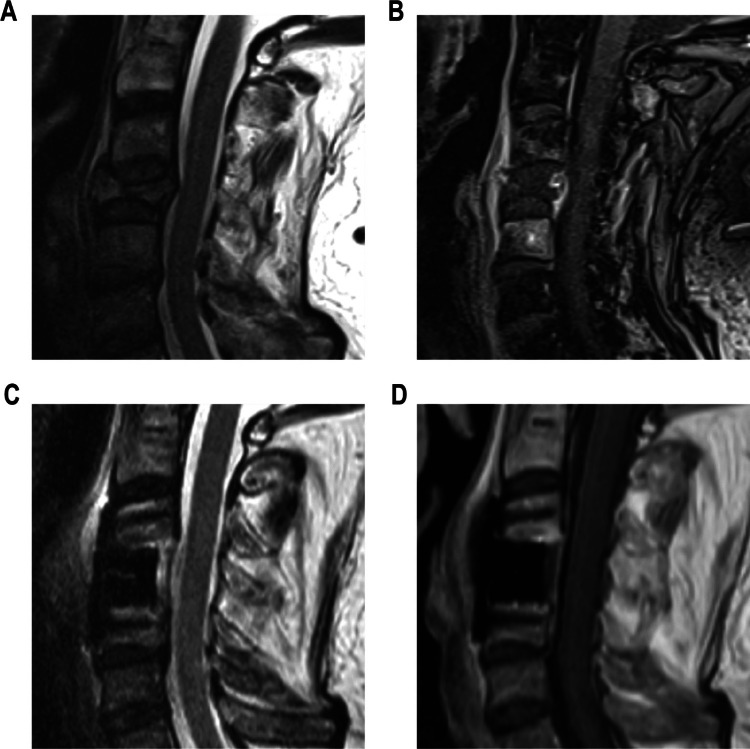
Case 2: Pre- (**A**, **B**) and postoperative (**C**, **D**) sagittal MRI T2 (**A**, **C**) and contrast enhanced T1 weighted imagesi (**B**, **D**)

A 33-year-old female patient with a known diagnosis of invasive ductal breast carcinoma (initial diagnosis in 2022) presented with severe, immobilizing neck pain without radicular radiation. Staging MRI revealed diffuse osseous metastases with a compression fracture of C4 and osteolytic destruction of C4 and C5, resulting in significant spinal canal stenosis (Fig. 4A, B).

Due to pronounced spinal instability and the risk of neurological deterioration, emergency surgical intervention was indicated. The patient underwent unilateral anterior corpectomy with vertebral body replacement at C4 and anterior cervical plating from C3 to C5 to restore spinal stability and decompress the spinal canal.

Postoperatively, the patient developed a marked left-sided deltoid paresis. Emergency postoperative MRI (Fig. 4C, D) ruled out a compressive hematoma or implant-related complication. The patient gradually recovered, and the deltoid muscle function improved steadily over time. At long-term follow-up, the deltoid paresis had completely resolved, and the patient remained neurologically stable.

#### Case 3 – thoracic CFRP cage with postoperative tumor progression

**Fig. 5:**
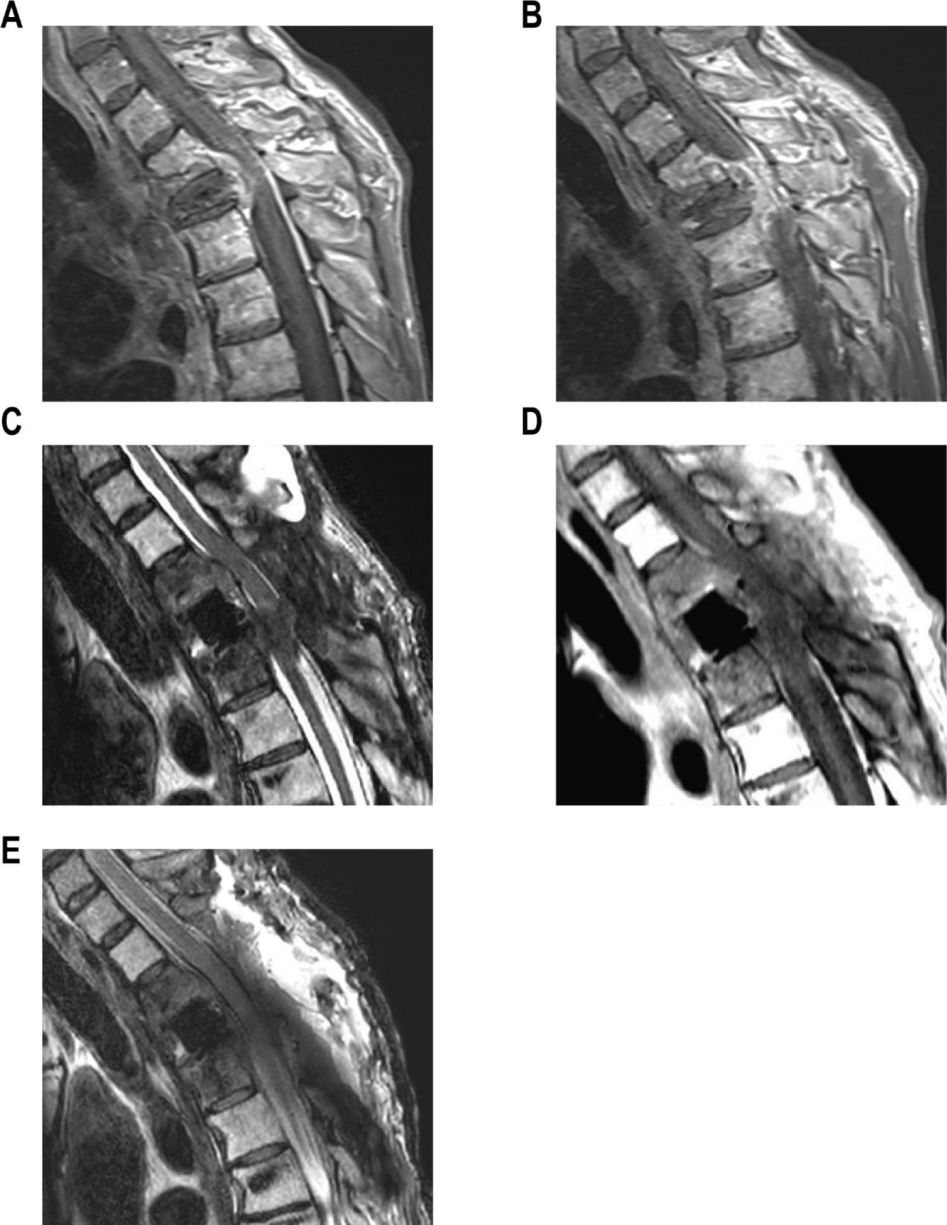
Case 3: Pre- (**A**, **B**) and postoperative (**C**, **D**,** E**) sagittal MRI T2 weighting (**A**, **C**, **E**) and T1 with KM weighting (**B**, **D**)

55-year-old male patient under oncological care for a sarcomatoid tumor presented with an incomplete spinal cord syndrome (ASIA D) with severe spinal ataxia and hypoesthesia. Preoperative MRI revealed severe spinal cord compression T2–T4 due to a spinal tumor manifestation (Fig. 5A, B).

A two-stage surgical approach was performed including decompression of the spinal cord via laminectomy at T3, followed by posterior stabilization using a CFRP screw-rod system from T1 to T6. Posterolateral CFRP vertebral body replacement was performed in a second surgical step.

Three months postoperatively, the patient presented again with worsening of the spinal cord syndrome, now exhibiting severe paresis. Emergency MRI of the spine (Fig. 5C, D) showed tumor progression with recurrent severe spinal cord compression at T2–T4. Consequently, an extension of the laminectomy at T2 and T4 was performed, along with an extension of the posterior stabilization from C6 to T7 using CRFP pedicle screws for biomechanical support.

Postoperatively, the patient's pre-existing paresis showed initial improvement. A postoperative CT scan confirmed the correct positioning of the implants, and MRI demonstrated significant spinal cord decompression (Fig. 5E). The patient was discharged with persistent ataxia but was able to walk independently.

#### Case 4 – thoracic titanium cage

**Fig. 6:**
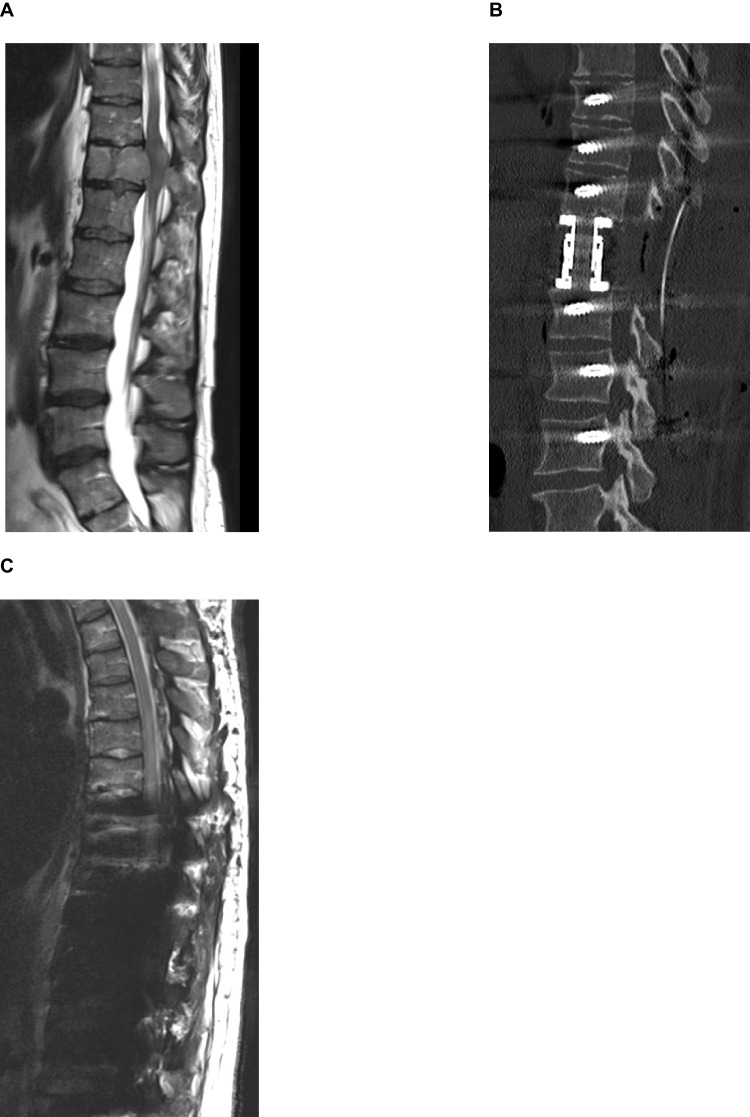
Case 4: **A** Preoperative sagittal T2-weighted images. **B** Postoperative CT confirming implant position, and follow-up sagittal T2-weighted MRI images (**C**)

A 47-year-old female patient presented with progressive lumbar back pain over several weeks. Spinal MRI revealed a homogeneously contrast-enhancing mass lesion occupying the entire vertebral body of Th11, with evidence of a pathological fracture, involvement of the posterior elements, and significant spinal canal compression (Fig. 6A). Urgent surgical intervention was indicated; however, the patient developed an acute complete paraplegia during the preoperative inpatient course.

Emergent MRI demonstrated an extensive epidural hematoma extending from T2 to T11. The patient was taken immediately to the operating room for posterior decompression and instrumentation. A posterior stabilization was performed using titanium pedicle screws at T8, T9, T10, T12, L1, and L2, followed in the same session by a lateral corpectomy of T11 and placement of a titanium cage (NuVasive XCore).

Postoperative CT confirmed correct positioning of all implants (Fig. 6B). Despite surgical intervention, the patient’s neurological deficits remained unchanged. Follow-up MRI was inconclusive due to metallic artifacts (Fig. 6C). Histopathological analysis of the lesion confirmed the diagnosis of a plasmocytoma. The patient was subsequently referred for oncologic treatment and transferred to a rehabilitation facility.

## Discussion

In this study, we present an institutional experience using CFPR vertebral body cages in oncologic spine surgery. We illustrate their functional comparability to titanium cages and demonstrate their advantages in assessing anatomical structures in postoperative MRI.

The use of radiolucent CFRP implants in spinal oncology marks a significant advancement in oncologic spine surgery, potentially enhancing patient outcomes by improving imaging interpretability, which could lead to better detection of postoperative complications and more effective radiotherapy planning [5, 7]. Several centers have increasingly used CFRP spinal implants for oncologic instrumentation in recent years [1, 2, 9, 11, 13, 15], as well as for other indications such as spondylodiscitis [4]. Hubertus et al. reported a low overall complication and revision rate for CFRP implants in a recent multi-institutional study [2], as well as a recent literature review [5], which showed comparable implant failure rates between CFRP and titanium implants (e.g., 1.7% vs. 2.4% fractures for CFRP vs. titanium pedicle screws), supporting the biomechanical equivalence of both.

The supposed advantages of CFRP spinal implants include superior visualization on postoperative MRI and CT, offering clearer delineation of adjacent soft tissues. This enhanced imaging enables surgeons to assess postoperative complications, such as bleeding or damage to neural structures, as well as monitor tumors during FU scans. In this regard, Krätzig et al. [6] analyzed the artifacts of CFRP screws compared to conventional titanium screws and demonstrated fewer artifacts on CT and MRI with CFRP screws, confirming these theoretical advantages.

The recent introduction of CFRP vertebral body implants has significantly expanded the range of available options, allowing even complex oncologic spine surgeries involving vertebral body replacement to benefit from the use of carbon-based implants. Several institutions have described the use of CFRP vertebral body cages in recent years [2, 13]. While Hubertus et al. reported in their multi-center experience the implantation of 101 CFRP vertebral body cages [2], they reported only 2 cage related postoperative complication which lead to revision surgery (1 CFRP and 1 titanium cage), Schwendner et al. reported implantation of 26 CFRP cages in 25 patients in the thoracic and lumbar spine [13]. In their study, two cases required revision surgery due to adjacent vertebral fractures with cage subsidence, but there were no failure of the implanted CFRP cages. Taken together, this overall comparable complication rate of CFRP to titanium cages is in line with our presented institutional experience. In addition, our study shows for the first time the improved assessability of different anatomical landmarks in CFRP cages in postoperative MRI. This complements the already described imaging advantages of CFRP screw implants [6] and implies the rationale for future studies regarding earlier detection of complications, tumor recurrence/progression or improved radiation planning. Overall, while our findings, along with the existing literature on this topic, suggest a potential benefit of CFRP cages for complication detection, tumor surveillance, and radiotherapy planning, these points should be addressed in future studies designed specifically to answer these questions before recommending the routine use of CFRP cages in spinal oncology patients, particularly given their higher costs and limited availability [16].

Taking together, there is growing evidence of improved postoperative imaging quality with CFRP implants, with complication rates comparable to those of titanium implants, including vertebral body cages, as demonstrated in our current study. However, future research should assess whether this leads to earlier detection of complications, improved radiotherapy planning, or earlier identification of tumor recurrences, ultimately improving patient care.

### Limitations

Certain limitations of this study should be acknowledged: This work reflects an institutional experience with a small series of CFRP vertebral body cages in oncologic spine surgery. In addition to the small sample size, the retrospective nature of the study could introduce selection bias, limiting the generalizability of our results. Although our study is the first to provide a postoperative MRI assessability analysis of CFRP compared to titanium cages, the scoring method used is subjective, despite the reported low inter-rater variability in our results, and no standardized postoperative imaging protocols were performed and therefore should be interpreted with caution.

## Conclusions

CFRP cages appear to be a promising alternative to conventional titanium cages in oncologic spine surgery. Our series suggests that they are functionally comparable to titanium cages, can be implanted in all spinal segments, and offer advantages in postoperative imaging. However, the benefit of this improved image quality—such as in postoperative complication detection, tumor surveillance, or radiotherapy planning—needs to be further explored in future studies.

## Supplementary Information

Below is the link to the electronic supplementary material.

ESM 1 Supplementary Material 1 (DOCX 181 KB)

## Data Availability

The anonymized data used in the analyses is available upon request.
