# Supplementary material for: Use of carbon fiber-reinforced PEEK cages in spinal oncology patients: An institutional experience with emphasis on surgical, complication and imaging characteristics
Source: Acta Neurochir (Wien). 2025 Dec 20;167(1):331. doi: 10.1007/s00701-025-06739-6 (PMC12722265; doi:10.1007/s00701-025-06739-6)
Supplement: Supplementary file 1 — Supplementary Material 1 (DOCX 181 KB) [file 701_2025_6739_MOESM1_ESM.docx]

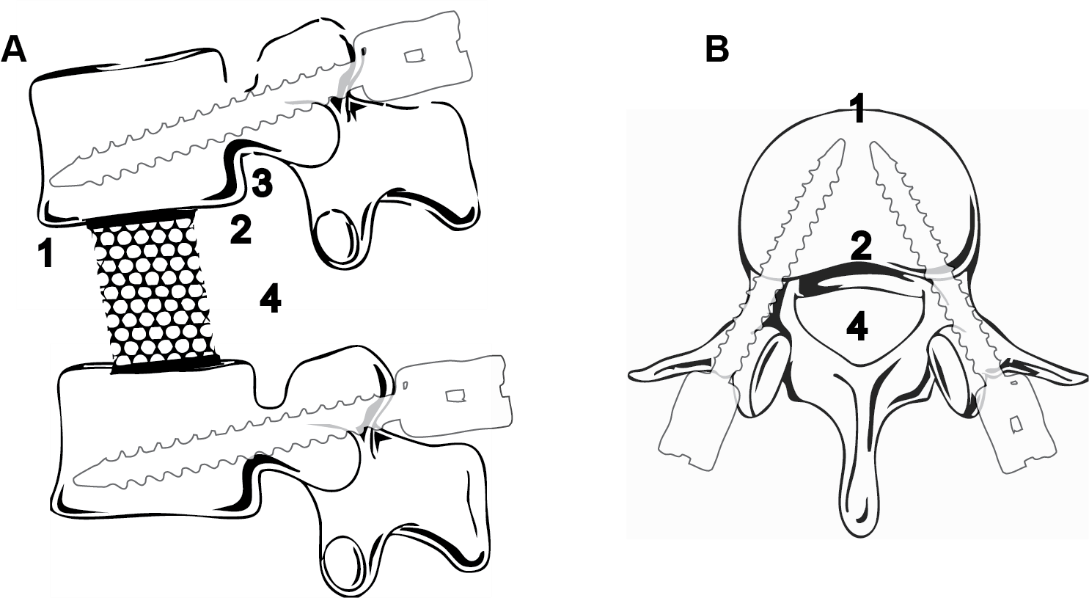


**Supplementary Figure 1:** Shematic drawing of the anatomical landmarks scored in sagittal (A) and axial (B) view. 1. Anterior vertebral column. 2. Posterior vertebral column. 3. Neuroforamen. 4 Spinal canal.

Adapted from: Servier Medical Art (license: https://creativecommons.org/licenses/by/4.0/)
